# Carcinoma of the Tongue Presenting as Trigeminal Neuralgia: A Case Report

**DOI:** 10.7759/cureus.20514

**Published:** 2021-12-19

**Authors:** David Kim, Adem Idrizi, Walaa Housny

**Affiliations:** 1 Anesthesiology, State University of New York Downstate Health Sciences University, Brooklyn, USA; 2 Anesthesiology, Brookdale University Hospital Medical Center, Brooklyn, USA

**Keywords:** contrast enhanced ct, lymphadenopathy, palliative management, chronic pain management, oral cancers, trigeminal nerve block, trigeminal nerve pain, trigeminal neuralgia, squamous cell carcinoma (scc)

## Abstract

Trigeminal neuralgia (TN) presents with extreme pain involving one or more branches of the trigeminal nerve (CN V). Although the exact cause of TN is still unknown, most cases have been linked to neurovascular compression of the nerve at the base of the brain. Pain refractory to medications can be treated with an image-guided trigeminal nerve block. A mass compressing on the trigeminal nerve can also present in rare cases of TN. Appropriate imaging is necessary to identify the likely cause of TN and develop a treatment plan prior to any intervention. We discuss the case of an 81-year-old woman diagnosed with invasive oral squamous cell carcinoma presenting as TN.

## Introduction

Trigeminal neuralgia (TN), also known as tic douloureux, is described as an excruciating pain involving mostly the lower face and jaw, although sometimes affecting the area around the nose and above the eye [1]. Classic TN is characterized by recurrent, stabbing pain involving one or more branches of the trigeminal nerve (CN V) [2]. The pain is usually unilateral; however, it may present as bilateral in rare cases. Neurovascular compression has been postulated as the main etiology of classic TN. Oral cancer as a cause of TN in patients is less common. In this report, we present a case of invasive lingual squamous cell carcinoma presenting as TN in an elderly patient.

## Case presentation

An 81-year-old woman with a known history of hypertension and chronic obstructive pulmonary disease presented to the emergency department with unrelenting left-sided facial pain and was ultimately admitted for severe TN. Her pain had started four months ago. She described it as a stabbing sensation that started from her left ear and radiated down her left jaw and tongue. She experienced around 30 painful episodes throughout each day, with each episode lasting a few minutes. Her pain mostly occurred sporadically, but she did notice that chewing triggered the onset of her pain. She rated her pain a 10/10 during her episodes with no relieving factors. She also had associated dysphagia, generalized weakness, and a 25-pound weight loss over the past four months. She had undergone a prior MRI head with pre- and post-gadolinium-based contrast agents (GBCA) two months ago, which had not shown any significant findings. Her initial medication regimen had consisted of carbamazepine, pregabalin, and acetaminophen/codeine, which had been later switched to trials of lamotrigine, pregabalin, bupropion, and fentanyl patches. Pharmacologic therapy failed to provide any relief. Initial physical exam was significant for hypotension with systolic blood pressures in the 90s. Her labs were significant for hypokalemia (2.3 mmol/L), hypomagnesemia (1.5 mg/dL), hypoalbuminemia (2.0 g/dL), and leukocytosis (16,000/mm³).

**Figure 1:**
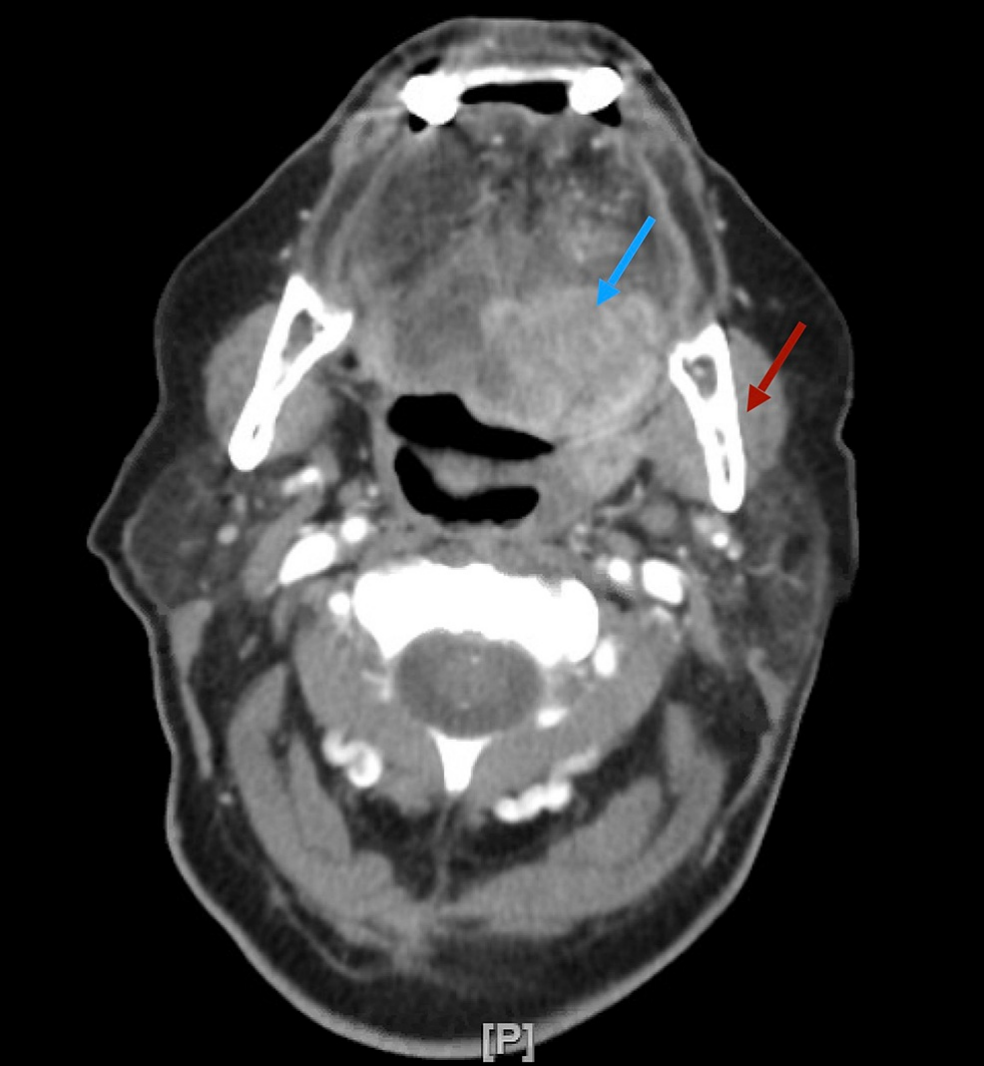
CT neck with contrast of an irregular, peripherally enhancing mass within the left sublingual (blue arrow) and submandibular space (red arrow) measuring up to 4.7 cm CT: computed tomography

During her hospital stay, the pain management service was consulted to evaluate the patient for a trigeminal nerve block. On physical examination, she was noted to have left-sided allodynia and hyperalgesia along the lower face with left-sided lymphadenopathy in the neck. A CT neck with contrast was ordered, which showed an irregular, peripherally enhancing mass within the left sublingual and submandibular space measuring up to 4.7 cm with necrotic non-enlarged bilateral level 2A lymph nodes (Figure 1). Due to suspicion for neoplasm, a biopsy was performed, which was positive for squamous cell carcinoma. The patient was diagnosed with T4N1 squamous cell carcinoma of the base of the tongue with the involvement of the extrinsic muscles of the tongue. Further imaging of the body was done to rule out metastatic disease. She was evaluated by radiation oncology to start radiation and chemotherapy.

While awaiting treatment, the patient developed acute respiratory distress requiring immediate intubation. She became pulseless, was made Do Not Resuscitate/Do Not Intubate (DNR/DNI) by the family, and was pronounced deceased. She was hypothesized to have had a massive pulmonary embolism given her risk factors for thrombosis, including being bedbound from lack of energy due to poor oral intake and having cancer.

## Discussion

TN, a rare debilitating disease characterized by lancinating facial pain, affects 4-13 people per 100,000 population [3]. In the United States, the incidence of TN is higher in women and increases with age [4]. Neurovascular compression of affected nerves is responsible for 80-90% of TN cases [5]. A proper workup of trigeminal nerve pathology should include a complete clinical history and physical examination and may include head imaging and electrophysiologic testing. The first-line treatment includes pharmacologic therapy with carbamazepine or oxcarbazepine, and if the patient is refractory to medical treatment, surgery is often warranted [6]. Additionally, reports have shown effective analgesia using ultrasound-guided trigeminal nerve blocks, which we employ at our pain management clinic for idiopathic TN [7,8].

In 15% of patients, painful trigeminal neuropathy is caused by multiple sclerosis or benign tumors of the cerebellopontine angle [6]. Previous cases have reported several tumors as causes of painful trigeminal neuropathy with most of these tumors being located in the posterior fossa [9,10]. One study has reported two cases of perineural metastasis of squamous cell carcinoma of the head and neck. Both patients had worsening facial pain over the years with additional cranial neuropathies with subsequent contrast MRI revealing intracranial enhancements [11]. Another report has described a 37-year-old woman with a rare brainstem schwannoma causing left-sided trigeminal neuropathy [12]. One study has reported a middle-aged male patient presenting with symptoms of atypical TN, including burning pain and sensory loss over the right temporal area and the tongue. Radiologic evaluation using contrast MRI showed a squamous cell carcinoma in the parapharyngeal space. A right V3 root block via an infra-zygomatic approach was performed and the patient was scheduled for radiation and chemotherapy [13]. A recent report has highlighted an unusual presentation of tongue lymphoma that had caused the patient to have a 10-month struggle with paresthesia and pain [14]. One report has highlighted the use of stereotactic radiosurgery for the treatment of TN in a patient, but the radiation ultimately led to oral carcinoma that presented with trigeminal neuropathy years later [15]. Interestingly, perineural invasion of periorbital squamous cell carcinoma presenting as trigeminal neuropathy has also been reported [16]. These reports indicate the necessity for extensive considerations and workups to accurately diagnose and treat painful trigeminal neuropathy due to cancerous etiologies.

Our case was unique and had significant features. Our patient’s chronic debilitating pain, which was refractory to pharmacologic therapy, resulted in poor oral intake, weight loss, severe hypokalemia (2.3 mmol/L), hypomagnesemia (1.5 mg/dL), and upon examination, left-sided lymphadenopathy and dysphagia were noted. This prompted radiologic imaging via CT neck with contrast, which revealed advanced invasive squamous cell carcinoma of the tongue with the involvement of the extrinsic tongue muscles located near the mandibular branch of CN V, likely causing nerve compression. Special considerations need to be assessed when considering an image-guided trigeminal nerve block on such patients as a palliative measure. An image-guided technique that avoids contact with squamous cell carcinoma is ideal in this patient population.

## Conclusions

Advanced lingual cancer with extrinsic muscle involvement can present as secondary TN, in addition to other symptoms. We encourage pain management specialists to use imaging modalities that include anatomical regions outside the brain for uncommon etiologies of TN. This can facilitate proper management and treatment of secondary TN unresponsive to drug therapy.
